# Error Modelling for Multi-Sensor Measurements in Infrastructure-Free Indoor Navigation

**DOI:** 10.3390/s18020590

**Published:** 2018-02-14

**Authors:** Laura Ruotsalainen, Martti Kirkko-Jaakkola, Jesperi Rantanen, Maija Mäkelä

**Affiliations:** Finnish Geospatial Research Institute (FGI), Geodeetinrinne 2, 02430 Masala, Finland; martti.kirkko-jaakkola@nls.fi (M.K.-J.); jesperi.rantanen@nls.fi (J.R.); maija.makela@nls.fi (M.M.)

**Keywords:** error modelling, sensor fusion, indoor positioning, particle filtering

## Abstract

The long-term objective of our research is to develop a method for infrastructure-free simultaneous localization and mapping (SLAM) and context recognition for tactical situational awareness. Localization will be realized by propagating motion measurements obtained using a monocular camera, a foot-mounted Inertial Measurement Unit (IMU), sonar, and a barometer. Due to the size and weight requirements set by tactical applications, Micro-Electro-Mechanical (MEMS) sensors will be used. However, MEMS sensors suffer from biases and drift errors that may substantially decrease the position accuracy. Therefore, sophisticated error modelling and implementation of integration algorithms are key for providing a viable result. Algorithms used for multi-sensor fusion have traditionally been different versions of Kalman filters. However, Kalman filters are based on the assumptions that the state propagation and measurement models are linear with additive Gaussian noise. Neither of the assumptions is correct for tactical applications, especially for dismounted soldiers, or rescue personnel. Therefore, error modelling and implementation of advanced fusion algorithms are essential for providing a viable result. Our approach is to use particle filtering (PF), which is a sophisticated option for integrating measurements emerging from pedestrian motion having non-Gaussian error characteristics. This paper discusses the statistical modelling of the measurement errors from inertial sensors and vision based heading and translation measurements to include the correct error probability density functions (pdf) in the particle filter implementation. Then, model fitting is used to verify the pdfs of the measurement errors. Based on the deduced error models of the measurements, particle filtering method is developed to fuse all this information, where the weights of each particle are computed based on the specific models derived. The performance of the developed method is tested via two experiments, one at a university’s premises and another in realistic tactical conditions. The results show significant improvement on the horizontal localization when the measurement errors are carefully modelled and their inclusion into the particle filtering implementation correctly realized.

## 1. Introduction

Many applications require rapid, accurate and reliable information about unknown indoor environments. Our research aims at providing the users with accurate and reliable infrastructure-free Simultaneous Localization and Mapping (SLAM) [1,2,3]. Infrastructure-free in this context means that the system is able to localize itself independent of any equipment pre-installed to the building. The localization solution will be based on fusing measurements obtained from multiple sensors carried by the user. Our final solution is a particle filter implementation fusing measurements from visual perception, a foot-mounted Inertial Measurement Unit (IMU), a barometer and sonar to obtain a three-dimensional SLAM solution [4]. In this paper we discuss the horizontal localization solution obtained using the IMU and visual perception. The vertical positioning solution was discussed in more detail in e.g., [5]. 

Traditionally sensor-based localization has been implemented by propagating a known initial position and heading with heading change and translation measurements obtained using an IMU. However, the MEMS sensors suffer from biases and drift errors that may decrease the position accuracy substantially [6]. Although mounting the IMU to the foot of the user [7] reduces errors substantially, especially high dynamics deteriorate the solution fast. Motion measurements, namely heading change and translation, computed from consecutive images provide a complementary positioning mean for an IMU. The two methods fused in our solution, namely foot-mounted pedestrian dead reckoning (PDR) and visual perception, have different error sources and therefore, when carefully fused, calibrate each other continuously. However, the localization solution deteriorates in time and sophisticated implementation of fusion algorithm is the key for improving the result.

A universal solution for infrastructure-free indoor localization remains still unsolved, but is an active research area. Many sophisticated solutions have been developed for indoor localization, but they mainly fuse inertial navigation with technologies requiring infrastructure, such as WLAN signals [8], or Ultra-Wide Band (UWB) beacons attached to e.g., construction site [9]. However, for tactical and rescue operations the requirement for using localization only in environments that are prepared for localization is too restrictive. Thereby, in our research we tackle the challenge by using only sensors which can be carried by the user and therefore allow localization anywhere. Reference [10] discusses an infrastructure-free positioning system using inertial navigation and magnetometric measurements and resulting in 6% positioning error in indoor mall walking experiments. However, tactical and rescue applications include often rapid and unusual motions, which set challenges for the localization system. 

Algorithms used for sensor fusion have traditionally been different versions of Kalman filters [11]. However, Kalman filters are based on the assumptions that the state propagation and measurement models are linear with additive Gaussian noise. Neither of the assumptions is correct for pedestrian applications, especially not for dismounted soldiers nor rescue personnel who are the two user groups significantly benefitting from the infrastructure-free SLAM implementation. 

Particle filtering is a sophisticated option for integrating measurements emerging from pedestrian motion [12]. These measurements do not usually have Gaussian error characteristics and therefore particle filtering provides best performance when the measurement errors are modelled and the correct probability density functions (pdf) introduced into the filter. Particle filtering is a Monte Carlo method, and in addition to the state transition and measurement models, the choice of sampling algorithms can affect the behavior of the filter [13,14,15]. In this work, we use the bootstrap filter [16]; this algorithm is popular because of its simplicity, but the tradeoff is the need for an increased amount of Monte Carlo samples for accurate state estimation.

In this paper we first discuss our measurement models and then the statistical modelling of the measurement errors and their most probable pdfs. Then, we present the particle filter implementation and experimental results for horizontal navigation using a dataset collected at two indoor data campaigns. The developed fusion method is compared to a particle filtering implementation, where the errors are considered to be Gaussian distributed. Finally, we give conclusions of the research and discuss further work.

## 2. Motion Measurements

Our solution for horizontal localization fuses heading change and translation measurements computed using a foot-mounted IMU and obtained via visual perception. Below the basics of both methods are discussed.

### 2.1. Foot-Mounted PDR

Using a conventional strapdown IMU specific force (**f**) and angular rate (*ω*) measurements corresponding to the IMU’s body coordinate frame (*b*) are observed. A strapdown inertial navigation solution can be obtained by solving the system of differential equations [16]. When the initial position, velocity, and attitude are known the obtained measurements can be used to propagate the position estimation in time.

In general, the low-cost MEMS inertial sensors introduce errors into the measurements and therefore are inadequate for use alone in the traditional strapdown mechanization except for very short periods of time. However, mounting the IMU to the foot of a pedestrian constitutes a special case; unless the shoe slips, the IMU remains stationary for a short period of time between steps and this fact can be used for committing zero velocity updates (ZUPT) for correcting some of the errors [17,18]. Therefore, the strapdown mechanization can essentially be interpreted as a boundary value problem instead of an initial value problem and the step displacement may be computed using:

(1) $$\{\begin{array}{c} \dot{C}=C\left(\omega^{b}\times \right)\\ {\dot{v}}^{n}=Cf+g^{n}\\ {\dot{p}}^{n}=v^{n} \end{array}$$

where the matrix *C* denotes the direction cosine matrix between the body frame *b* and the navigation coordinate frame *n*; × denotes the 3 × 3 skew-symmetric cross product matrix; $v^{n}$ and $p^{n}$ are the velocity and position in the navigation frame, respectively; and $g^{n}$ denotes the local gravitational acceleration vector. Note that these equations neglect certain factors that are not significant in pedestrian navigation such as the transport rate and the Coriolis force [16].

The strapdown mechanization is implemented with the help of an error-state Kalman filter modeling the propagation of position, velocity, and attitude errors. In foot-mounted PDR it is straightforward to apply a ZUPT to the error-state filter whenever the IMU is detected to be at rest. This way improved displacement and heading measurements are obtained. The measurements observed also justify the use of the term PDR in the context of the strapdown navigation although traditionally PDR and strapdown mechanization have been used exclusively [19]. However, note that the displacement measurement in foot-mounted PDR is a three-dimensional vector instead of a scalar quantity. A similar dead reckoning approach has been chosen in, e.g., [20]. 

Inertial measurement errors consist of a constant part, which may be determined by calibration, and a stochastic part constituting random errors [21]. The constant part has been obtained from the calibration report provided by the instrument manufacturer and literature, and in our method it has been incorporated into the strapdown navigation computations. The random errors originate mainly from temperature changes that affect especially the performance of the gyroscope [22] and the dynamics that induce e.g., G-sensitivity errors [23]. These random errors must be modelled for improved localization solution and the process is discussed in Section 3.

### 2.2. Visual Navigation

Motion of features in consecutive images provides heading change and displacement information when the camera is attached to the user body. In our earlier research, we have presented concepts called visual gyroscope and visual odometer [24], which provide measurements allowing a camera to be considered as an additional self-contained sensor. 

The visual gyroscope concept is based on the fact that most human-made environments, like indoors and urban areas, consist of rectilinear features and planes aligned in three orthogonal directions. The edges of these elements can be seen as lines that seem to intersect in a point called a vanishing point. Changes of the locations of the vanishing points are defined by the orientation of the camera with respect to the scene. Therefore, when the changes of vanishing point locations are tracked the change in the camera orientation may be observed. When the camera is attached to the user body, the change in the camera heading may be conceived as the users heading change. 

Due to the size and accuracy requirements, monocular cameras have advantages for visual perception compared to stereo cameras [25]. However, the difficulty in resolving the distance between the camera and a landmark tracked in the consecutive images induces an ambiguous scale into the translation. So far the best vision-only solution for resolving this challenge and therefore obtaining the real metric translation, has been the use of a homography formula [26], applicable when tracking the image features of objects found on a ground plane. Our implementation of a visual odometer is based on the homography formula using only the knowledge of the camera height as a pre-requirement. The visual odometer observes the orientation of the camera using the visual gyroscope presented above. 

Errors in the visual perception arise mainly from the environmental reasons. For visual gyroscope, the most crucial reasons are that there are not enough lines in the scene or the lines are non-orthogonal. Although the visual gyroscope is quite robust despite changes in lighting and camera dynamics, errors deteriorate occasionally the measurements. In our earlier research, an error detection method called Line Dilution of Precision (LDOP) [27] has been developed. LDOP is a value based on the geometry of lines used for the vanishing point computation. When the line geometry is observed to be poor, the measurements are discarded. Also, the translation measurements obtained using the visual odometer deteriorate from tracking erroneously matched image points. Therefore, Random Sample Consensus (RANSAC) [28] algorithm is used for improving the matching result.

Despite careful detection, errors always remain and therefore their probability distribution should be modelled in order to obtain an accurate fused localization solution.

## 3. Error Modelling

**Figure 1:**
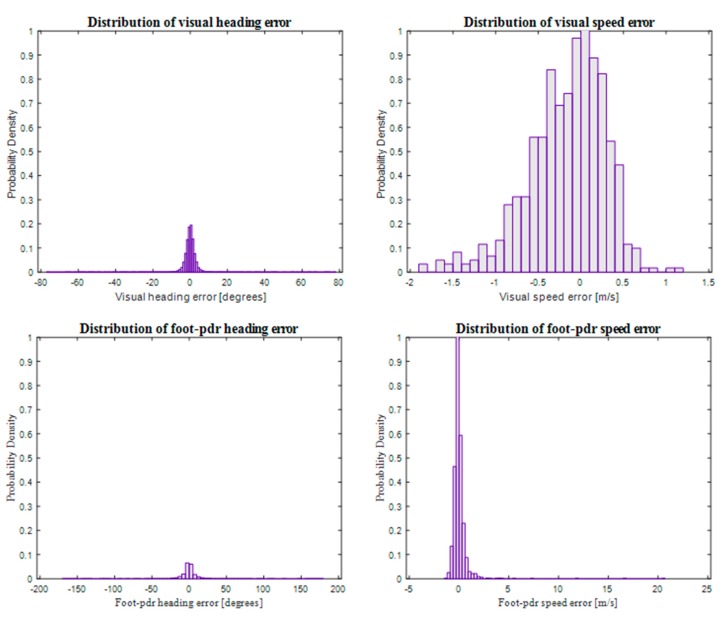
Probability density functions for visual heading, visual speed, foot-PDR heading and foot-PDR speed measurement errors.

As discussed above, measurement errors in pedestrian navigation do not usually have a Gaussian distribution. As presented in Figure 1, this is the case also in our setup. The data used for our navigation solution, namely the heading and speed measurements produced using visual perception and foot-PDR are far from being normally distributed. Heading measurement errors using both measuring techniques, as well as foot-PDR speed measurement errors, follow heavy-tailed distributions and the visual speed measurement error seems to have a bias. The statistical mean errors of the measurements are 0.035 degrees for visual heading change, −0.2122 m/s for visual speed, 0.1378 degrees for foot-PDR heading and 0.0102 m/s for speed. Therefore, data fusion using Kalman filtering does not produce an optimal solution. Particle filtering, discussed more in the following section, provides means for introducing the correct probability density functions (pdf) into the filter and thereby improving the accuracy of the obtained localization solution.

In order to introduce the correct error models into the fusion algorithm, we have done statistical modelling using data collected in various navigation experiments in different indoor environments. We use the method of Maximum Likelihood to find the correct distribution parameters. A statistic used to approximate a parameter *θ* is called a point estimator for *θ* and is denoted $\hat{\theta }$. In our approach, we compute the Maximum Likelihood Estimator (MLE) $\hat{\theta }$ for all probability distributions suitable for presenting our data, and then calculate the goodness-of-fit characteristics of each distribution with corresponding parameter estimators using Chi-Square test. The distribution with its parameter estimators obtaining the best goodness-of-fit result is then selected as the correct error distribution for each measurement in our method. Below the MLE method and Chi-Square goodness-of-fit methods are briefly discussed. Then, the results of our error modelling for foot-mounted PDR and visual heading and speed measurements are presented.

The MLE method is summarized as follows [29]. First, we define a distribution of a random variable *x*, which has the density of *f* and a parameter *θ*. Then, we obtain a random sample *x*_1_,…,*x_n_* with *n* observations from the distribution and define a likelihood function L(*θ*) as:

(2) $$L\left(\theta \right)=\prod_{i=1}^{n}f(x_{i}).$$

**Figure 2:**
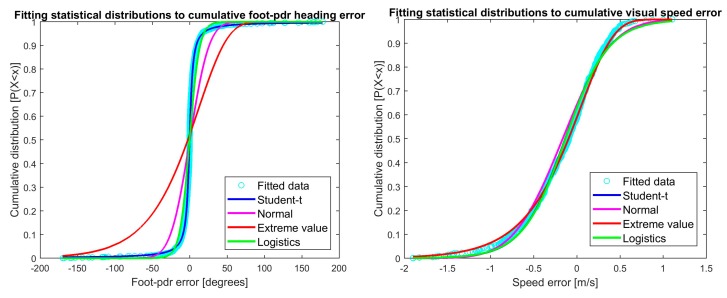
Cumulative distribution function plots for selected distributions for visual heading and speed errors. Turquoise is the real error data distribution and other plots data fitted to the selected distributions and parameters MLEs.

**Figure 3:**
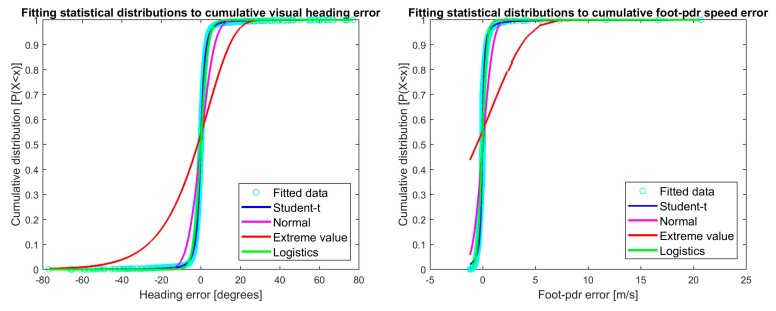
Cumulative distribution function plots for selected distributions for foot-PDR heading and speed errors. Turquoise is the real error data distribution and other plots data fitted to the selected distributions and parameters MLEs.

The MLE estimator $\hat{\theta }$ is now the parameter *θ* maximizing the likelihood function *L*(*θ*). Figure 2 shows the cumulative distribution functions plotted for real error data distribution and data fitted to the selected distributions with parameter MLEs for visual heading and speed errors, and Figure 3 the corresponding plots for foot-PDR. The fitted distributions are Student’s t location-scale, Normal, Extreme value and Logistic distributions. Hints of the best fitting distributions may be seen already from the figures, but will be statistically estimated below.

The goodness of fit of each distribution and corresponding parameter estimates is evaluated with a Chi-Square goodness-of-fit test (Chi2-gof) [29]. The Chi2-gof tests if the set of observations is drawn from the specified probability distribution. If *n* is large, the random variable:

(3) $$\sum_{i=1}^{k}\frac{{\left(X_{i}-np_{i}\right)}^{2}}{np_{i}}$$

follows an approximate chi-squared distribution. *X*_1_,…,*X_k_* is a multinomial random variable with parameters *n*, *p*_1_,…,*p_k_*. The distribution has *k* − 1 degrees of freedom, meaning that there are *k* mutually exclusive categories of variables. The degree of freedom depends on the fitted probability distribution. The multinomial random variable *X_i_* is the observed measurement, we may denote *X_i_* by *O_i_*, the observed frequency, and therefore *np_i_* is the theoretical expected number of trials by *E_i_*, the expected frequency. Then, we may formulate (*X*) as:

(4) $$\sum_{i=1}^{k}\frac{{\left(O_{i}-E_{i}\right)}^{2}}{E_{i}}$$

which also follows the chi-squared distribution. From this form it is easy to see that if the observed and expected frequencies differ remarkably, (*X*) will be large, and the proposed distribution is not a good fit for the data. 

We committed the Chi2-gof test for all measurement errors using the selected distributions and the parameters estimated by MLE. Table 1 shows the results for distributions that Chi2-gof test shows to describe the data with 5% significance level, namely the null hypothesis that the data comes from the distribution cannot be rejected. The test statistic is as described in (4). *p*-Values also support the decisions. In case *p*-value would be under, or even close to the selected significance level (0.05) we would have to reject the null hypothesis or at least have significant doubt on its validity [30]. For the selected distributions this is not the case, Table 1 shows large *p*-values for all selected distributions.

The most fitting probability distribution for visual heading measurement error is the Logistic distribution, and the Extreme value for speed. Errors for both foot-PDR heading and speed are best represented using the t location-scale distribution. Figure 3 showed that both foot-PDR measurements had errors with heavy-tail distributions, namely Student’s t-distributions. However, standard Student’s t-distribution has zero mean and variance restricted by the obtained model. Therefore, t location-scale distribution, a location-scale extension of Student’s t-distribution describes the situation of observing the motion using a foot-PDR better by giving flexibility on the distribution parameters [31].

## 4. Bayesian and Particle Filtering

Particle filtering provides improved performance for a localization system fusing non-linear measurements corrupted by non-Gaussian errors. The drawback of particle filtering is its computation time, which may be tenfold, or even hundredfold [32] compared to Kalman filtering. The required computation time is dependent on the number of states and especially the number of particles introduced to the model. A compromise has to be done between accuracy and computation time requirements set for the system in real-life applications, especially in tactical ones. 

Monte Carlo is a stochastic sampling technique for producing results from analytically intractable systems [33]. Sequential Monte Carlo methods, particle filtering as one, combine Monte Carlo sampling methods with Bayesian estimation, however, this is also the reason for its computational burden. Particle filtering uses a number of independent random samples, particles, for estimating the system state. The posterior probability is represented with a probability density function *p(x_k_)* using particles $x_{k}^{\left(i\right)}$, *i* = 1,…,*N*, which are sampled from the state space:

(5) $$p\left(x\right)\approx \overset{N}{\underset{i=1}{\sum }}w^{\left(i\right)}\delta (x_{k}-x_{k}^{\left(i\right)}),$$

where δ(⋅) is the Dirac delta function and $w^{\left(i\right)}$ the particle weight. 

As in Kalman filtering, the algorithm consists of prediction and update steps, where posterior is updated using new observations. Generally, the particles are drawn using a so-called proposal distribution; the ideal proposal distribution would be the posterior state distribution [13], but this distribution is generally not known. Indeed, the purpose of the filter is to estimate the posterior distribution. In this article, we use the particle filter variant called the bootstrap filter [17] where the proposal distribution is chosen to be the same as the transitional distribution $p(x_{k}|x_{k-1})$. Since this algorithm does not utilize the information conveyed by the most recent measurement to construct the proposal distribution, one should expect to need a higher number of particles in contrast to algorithms such as the auxiliary particle filter [34] or other methods to approximate the posterior distribution [13]. In this work, the results were computed in post-mission processing; for real-time implementations with processing power and battery constraints, a filter variant that can cope with a smaller amount of particles would be highly recommendable.

*Prediction:* Every particle $\left(x_{k-1}^{\left(i\right)},w_{k-1}^{\left(i\right)}\right)$ of the last posteriori probability $p(x_{k-1}|z_{0:k-1})$ is represented by a new one according to the process model $p(x_{k}|x_{k-1})$, resulting in a new set of particles $\left(x_{m,k}^{\left(i\right)},w_{k-1}^{\left(i\right)}\right)$, which represents a priori pdf at time $t_{k}$.

*Update:* The weight $w_{k}^{\left(i\right)}$ of every particle of the priori pdf is updated according to the measurement model:

(6) $$w_{k}^{\left(i\right)}=w_{k-1}^{\left(i\right)}\cdot p(z_{k}{|x}_{m,k}^{\left(i\right)}),\;\sum_{i=1}^{N}w_{k}^{\left(i\right)}=1$$

The reweighted set of particles now approximates the posterior pdf $p(x_{k}|z_{0:k})$.

Unfortunately, sequential updates can lead to a situation where only a handful of the samples have a nonzero weight. This phenomenon is commonly known as degeneracy and can be solved by including an additional resampling step. In resampling a new set of particles is drawn with replacement from the previous set. The probability of a certain particle to be included into the new set is defined by its weight factor. The particles are equally weighted in the resulting posterior distribution. Thereby, during the resampling step, unlikely particles are discarded and replaced by more likely ones. 

In practice, the resampling logic described above increases the variance of the state estimate [13]. For this reason, various resampling algorithms with different properties have been proposed [15] as well as other means for mitigating degeneracy, such as the introduction of artificial dynamics [14]. In this work, we employ multinomial sampling whenever the effective number of particles falls below 20% of the total, with this threshold value chosen empirically. In addition to degeneracy, tuning the resampling criteria and algorithm can mitigate another problem known as sample impoverishment where the diversity among the set of particles is reduced to consist of multiple duplicates of a handful of different samples.

### Process and Measurement Models

As discussed above, the computation time of a Particle filter based fusion is dependent on the number of states included in the model. Therefore, our state vector (**x***_k_*) consists only of the three-dimensional position solution having *x*, *y* and Height (*H*) components and heading (*ψ*) being **x***_k_* = [*x y H ψ*]*_k_*^T^ and uses 1000 particles. The horizontal position components (*x* and *y*) are computed by fusing the above discussed foot-PDR speed (*S_foot_*_−*PDR,k*_) and heading change (${\dot{\psi }}_{foot-PDR,k}$) measurements with the visual gyroscope’s heading change (${\dot{\psi }}_{visual,k}$) and visual odometer’s speed ($S_{visual,k}$) measurements. Vertical position component (*H*) is computed by processing the barometer and sonar measurements discussed in detail in [5]. The measurement model is:

(7) $$\begin{array}{c} \begin{array}{c} {\dot{\psi }}_{visual,k}={\dot{\psi }}_{k}+v_{1,k}\\ S_{visual,k}=S_{k}+v_{2,k}\\ {\dot{\psi }}_{foot-PDR,k}={\dot{\psi }}_{k}+v_{3,k} \end{array}\\ S_{foot-PDR,k}=S_{k}+v_{4,k}\\ \begin{array}{c} H_{baro,k}=H_{k}+v_{5,k}\\ H_{ultrasonar,k}=H_{k}+v_{6,k} \end{array} \end{array}$$

where $\dot{\psi }$ is the real heading change, *S_k_* speed and *H_k_* height. The measurement errors *v_n_*_,*k*_, *n* = 1.4 follow the error distributions discussed above, namely:

(8) $$\begin{array}{l} v_{1.k}\sim Logistic(0.004,1.77)\\ v_{2,k}\sim GEV(0.045,0.39)\\ v_{3,k}\sim tlocation-scale(-0.11,3.25,1.12)\\ v_{4,k}\sim tlocation-scale(-0.04,0.15,1.41) \end{array}$$

This paper addresses only the horizontal localization and therefore the errors deteriorating the vertical location were not modelled. The barometer and sonar errors are set as zero mean Gaussian noise, namely (*v*_5,*k*_) ~ N(0,σ_barometer_) and (*v*_6,*k*_) ~ N(0, σ_sonar_).

## 5. Experimental Results

The performance of our method was tested in two different experiments. The first experiment was done inside a university building, where a pedestrian, equipped with the sensors, walked an 880 m long route. The test person was carrying a reference system enabling the verification of the solution accuracy. However, infrastructure-free localization is mainly needed by first-responders, police and tactical applications, where experiments done by walking normally and carrying heavy reference system do not give realistic analysis of the method. Therefore, a proof-of-concept (PoC) experiment was carried out on military premises, in which a soldier acted as a test person and more challenging dynamics were experienced. The soldier was wearing real combat equipment in order to experience all real-life application-related motions. The setups for the two tests and the results are discussed below. 

### 5.1. Test Setup

**Figure 4:**
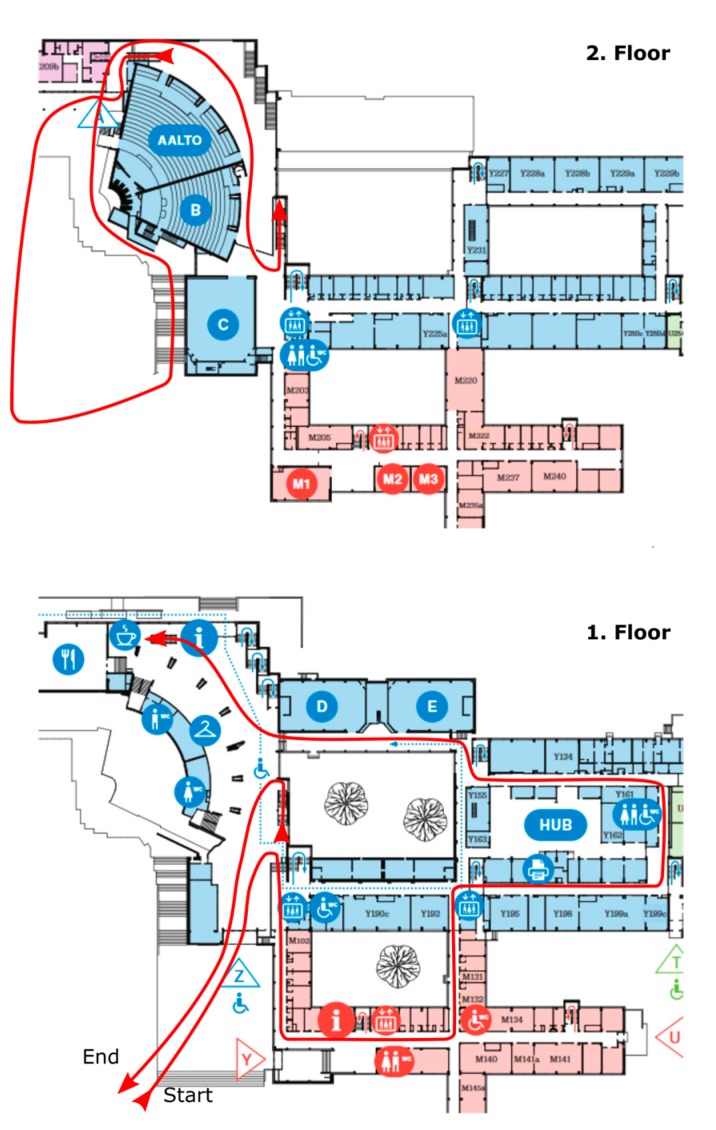
Floorplan of the university environment. The route travelled through two floors and is shown with a red line.

The test setup for both experiments consisted of one IMU attached to the foot of a test person and a camera on the shoulder. For the first experiment done at the university premises, the IMU was an Inertial Elements MIMU22BT (GT Silicon Pvt Ltd., Kanpur, India) and the camera a HERO5 Session (GoPro Inc., San Mateo, CA, USA). During the first experiment the test person carried also a SPAN system (NovAtel Inc., Calgary, AB, Canada) including a GNSS receiver with a HG1700 AG58 tactical grade IMU (Honeywell Aerospace, Phoenix, AZ, USA). The reference system provides decimeter-level accuracy for tens of minutes indoors and it was used to determine the real trajectory for assessing the performance of our fusion method. During the experiment an 880 m long route was walked using normal walking speed. The test environment consisted of short outdoor parts, indoors stairs, corridors and wider hall areas. The route travelled through two floors and is shown with a red line in Figure 4.

**Figure 5:**
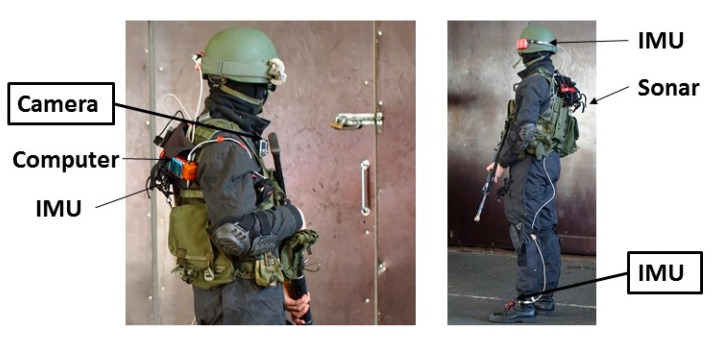
Setup for the proof-of-concept experiment done at the military premises by a soldier wearing combat equipment.

**Figure 6:**
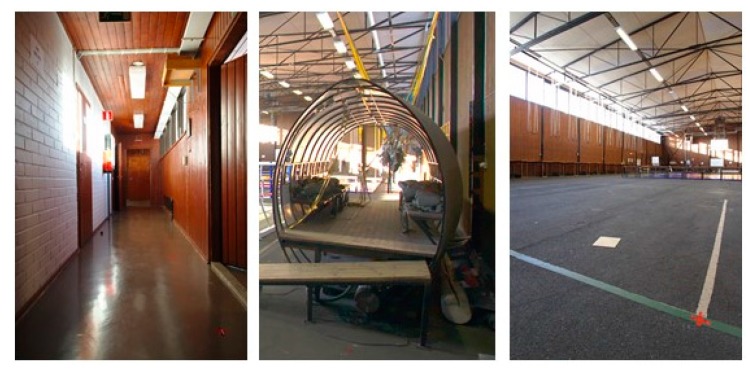
Selected pictures from the military PoC experiment showing the variation of the navigation environment.

Figure 5 shows the setup for the second experiment at the military premises. In addition to an MTw Awinda IMU (Xsens Technologies B.V., Enschede, The Netherlands) on the foot and SJCAM camera (Shenzhen Hongfeng Century Technology Co. Ltd., Shenzhen, China) on the shoulder, both used for computing the horizontal localization solution discussed herein, the soldiers carried other sensors that were not part of this experiment. The test done at the military premises was a proof-of-concept of the method in a real-life scenario. Therefore the test was done by a soldier and no reference system preventing the nominal dynamics in tactical applications was used, but the accuracy of the solution was evaluated based on the accuracy of the loop-closure at known start and end points. The route included dynamics which are challenging for both inertial sensing and visual perception, namely running, climbing, moving sideways and jumping up and down from constructions. The environment of the almost 400 m long route consisted of narrow corridors, small room and a wider hall, selected parts shown in Figure 6. The solution was computed by post processing the data for both experiments.

### 5.2. Results

The horizontal localization results for both experiments are presented below.

#### 5.2.1. Experiments at University Premises

**Figure 7:**
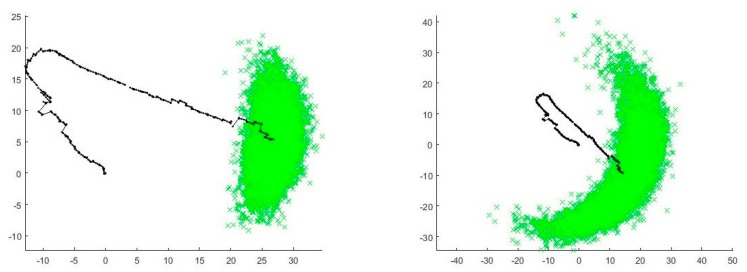
Distribution of particles (green) for foot-PDR particle filtering fusion position solution (path in black). Fusion computed using Gaussian distribution for foot-PDR measurement errors (on left) and t location-scale distribution (on right).

We computed the navigation solution using the particle filtering fusion method described above, incorporating all correct measurement error distributions presented in (8). The accuracy of the resulting fused horizontal localization solution was not satisfying, namely the Root Mean Squared Error (RMSE) was over 100 m. The reason for the fusion’s poor performance seemed to be the large but rare errors in foot-PDR measurements also visible in Figure 1. While analyzing the effect of the errors in the fusion filter we run the filter using 10,000 particles. The effect of the errors is clearly visible from the distribution of the particles. Figure 7 shows the dispersion of the particles when the foot-PDR measurements errors are incorporated to the filter as Gaussian (on left) and t location-scale (on right). When the measurements are fused with the assumption of Gaussian errors, the particles are concentrated and diverging particle clouds die out soon. T location-scale error distribution gives too much emphasize on the random, large but rare, foot-PDR errors and therefore the particle cloud diverges resulting in poor accuracy. Due to the emphasize given to the extreme errors, we deemed the foot-PDR error modelling results unusable for the practical particle filter implementation and therefore fitted the nominal Student’s t distributions for foot-PDR heading and speed errors as setting the *v_3,k_* and *v_4,k_* values in (8) as:

(9) $$\begin{array}{l} v_{3,k}\sim \begin{array}{cc} Student_{\prime }s & t \end{array}(7)\\ v_{4,k}\sim \begin{array}{cc} Student_{\prime }s & t \end{array}(3) \end{array}$$

**Figure 8:**
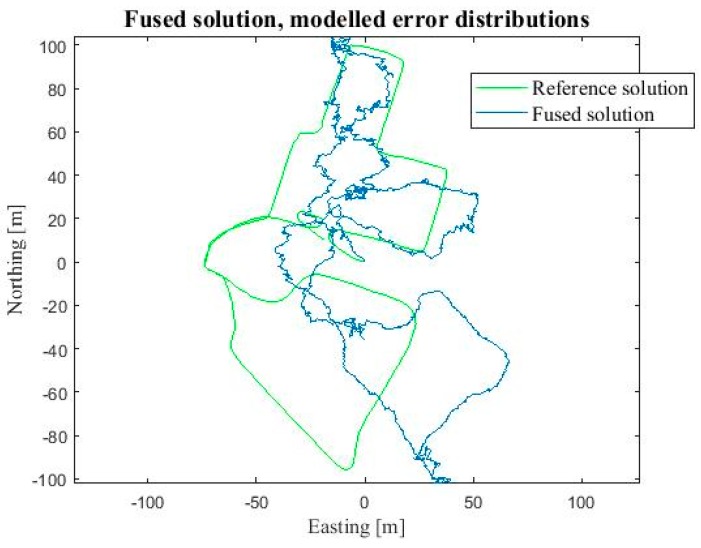
Horizontal position solution using the developed particle filter fusion algorithm and correct measurement error models (blue) and the reference solution (green).

The position result obtained using the Student’s t error distributions for the foot-PDR heading and speed measurements had RMSE 20.9 m, the Circular Error Probable (CEP), showing the error of which 50% of the obtained positions passed underneath, was 28.9 m and the 90 Percentile error (90% of positions passed underneath) was 56.3 m. The resulting route is shown in Figure 8. The result was still unsatisfying and therefore we computed the position solution using a particle filter implementation with visual errors modelled as discussed above, but foot-PDR errors estimated to be normally distributed:

(10) $$\begin{array}{l} v_{3,k}\sim N(0,\sigma_{foot-pdr\_heading})\\ v_{4,k}\sim N(0,\sigma_{foot-pdr\_speed}) \end{array}$$

**Figure 9:**
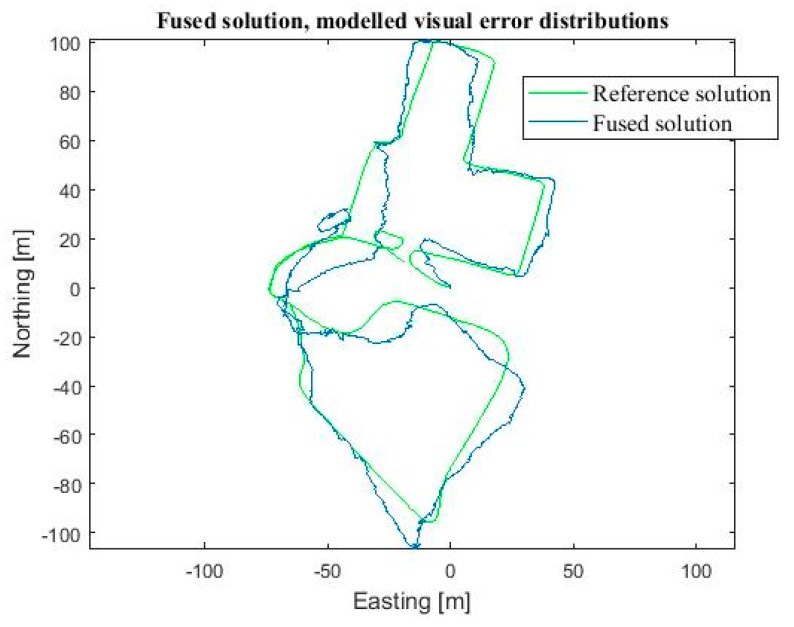
Horizontal position solution using the developed particle filter fusion algorithm and correct visual measurement error models (blue) and the reference solution (green).

**Figure 10:**
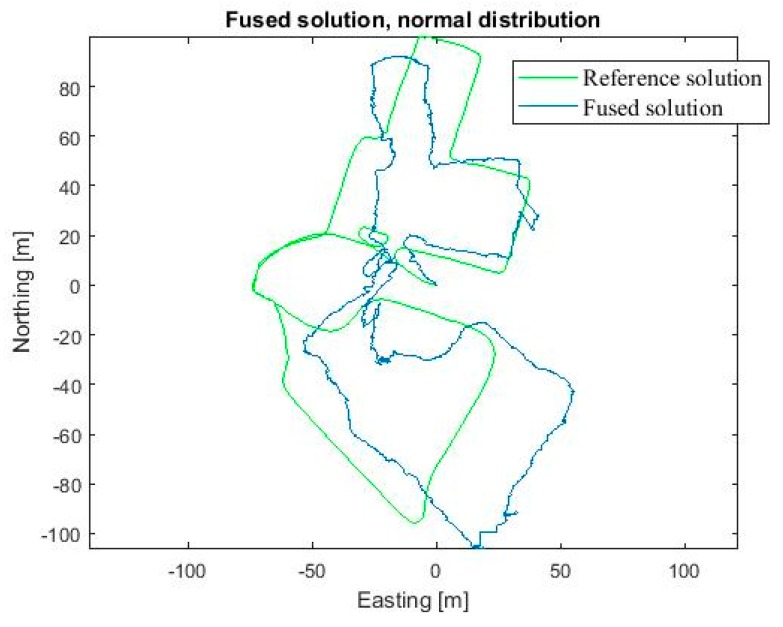
Horizontal position solution using the developed particle filter fusion algorithm using Gaussian measurement error models (blue) and the reference solution (green).

The location result using the final implementation was reasonably good as the Gaussian distribution neglected the extreme foot-PDR errors. The RMSE was 8.8 m, CEP 7.9 m and the 90 Percentile error 22.3 m. We computed the location also by using 10,000 particles, to evaluate the effect of the particle count on the accuracy and computation cost. With 10,000 particles the RMSE improved to 8.6 m, and 90 Percentile error to 21.8 m, and CEP was almost the same as for 1000 particles, 8.0 m. However, the computation time was tenfold, namely 320 s compared to 28 s with 1000 particles. Therefore, 1000 particles were evaluated to provide the best accuracy—computation time ratio. For comparison purposes, particle filtering fusion was done for the exactly same data using zero mean Gaussian error distributions also for visual measurements instead of the modelled error distributions. Using Gaussian distributions the accuracy of the position solution degraded remarkably, namely the corresponding errors were RMSE 16.3 m, CEP 25.8 m and 90 Percentile 44.7 m. Then, we computed the localization result using Gaussian modelling for all measurements, but this time we used the correct statistical mean errors presented in Section 3. The RMSE was reasonable good, 8.8 m, but the CEP and 90 percentile errors were notably worse than when using the real visual distributions, namely 9.7 m and 23.1 m, respectively. Table 2 summarizes the errors. Figure 9 shows the resulting position solution using the fusion method discussed (blue) and the reference solution (green). Figure 10 shows the corresponding fusion result when the Gaussian error distributions are used for all measurements in particle filtering (blue) and the reference solution (green). 

The results showed that by using the modelled visual heading and speed measurements, the accuracy of Particle filtering location solution was sufficient for infrastructure-free indoor localization. However, the nature of foot-PDR measurements including rare but large errors, required the errors to be modelled as Gaussian in the particle filtering implementation in order to not give them too much influence on the fusion performance. At least two factors have been observed to cause occasional large errors in foot-PDR. First, false zero-velocity detections can lead to a navigation filter update with erroneous information. Second, the utilized low-cost IMU and Raspberry Pi based data collection yielded in an uneven data sampling rate, with occasional notable data gaps. 

#### 5.2.2. Proof-Of-Concept Experiments at Military Premises

As discussed above, the proof-of-concept testing was committed in military premises and by a soldier wearing combat equipment. The route of 170 m was travelled two times, first by walking normally and then by running, resulting in 340 m long experiment. Both times the motion included also climbing up, down, and moving sideways on the wall bars, and jumping up and down from constructions. 

**Figure 11:**
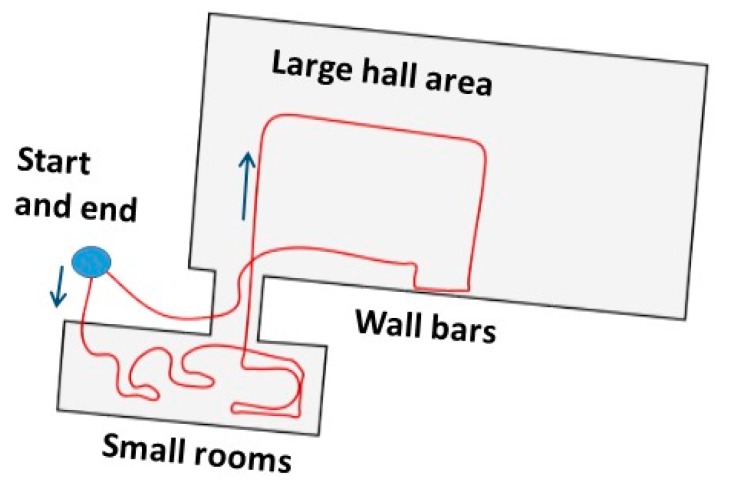
Route travelled during the POC experiments. The route is relative, drawn manually and not in scale.

Due to the lack of a reference solution the accuracy was computed as the error of the loop-closure, namely as the difference between the obtained route end-point and the known end-point. Figure 11 shows the relative route travelled. The route is drawn manually and is not to scale, but its purpose is to give an indication of the route. 

**Figure 12:**
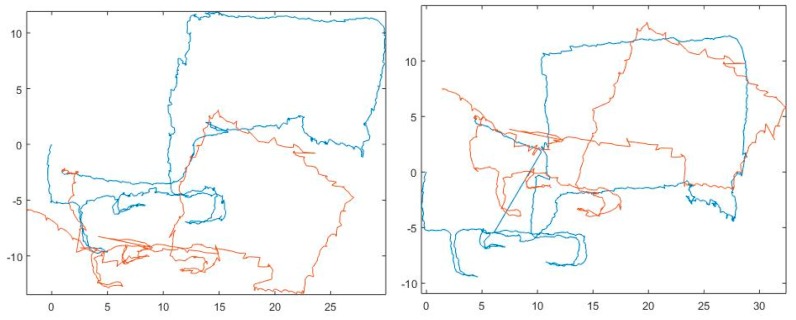
Horizontal location solution in the Proof-of-Concept (PoC) test. The fusion solutions are shown for experiments done by walking (blue) and running (red). On left the solution using the developed particle filter fusion algorithm with correct visual measurement error distributions and on right when using Gaussian measurement error models.

The localization solution was computed using the particle filtering fusion method discussed above. Due to the results obtained during the experiments discussed above, the filter was implemented by using the modelled error distributions for the visual heading and speed measurements, but the foot-PDR measurement errors were estimated to have a Gaussian distribution. The resulting navigation solution path is shown in Figure 12 (on left). For comparison the fusion was implemented by using Gaussian error distributions also for visual measurements. The resulting route is shown in Figure 12 (on right). As discussed above, the route was first travelled by walking (blue path in Figure 12) and then for a second time running (red path). Both times the motion included climbing, moving sideways, rapid turns and jumps. The error in loop closure was 2.54 m for the first round and 4.65 for the second. The location and direction were not calibrated between the routes. While using the Gaussian distributions for all measurements the corresponding errors were 6.53 m and 3.88 m. As a result, the developed particle filtering fusion method is shown to be feasible for infrastructure-free tactical indoor localization containing challenging dynamics and challenging environment. 

## 6. Discussion

The goal of our research is to develop a system for infrastructure-free tactical situational awareness. As the system has to be light, small and cost-effective, it requires the use of MEMS sensors. Measurements from MEMS sensors suffer from errors deteriorating the localization solution and therefore our solution is to develop particle filtering fusing measurements from multiple sensors. The horizontal location solution will exploit measurements from foot-mounted IMU and a body-mounted camera. First, we analyzed the error distributions of foot-PDR and visual heading and speed measurements and verified them not to follow a Gaussian distribution. This supports the decision of developing a particle filtering method for measurement fusion, as it allows the incorporation of the correct error models unlike Kalman filtering. Then, we modelled the error distributions by estimating the parameters with MLE and evaluating the goodness-of-fit of selected distributions using Chi-square testing. The performance of the particle filtering fusion was evaluated by two experiments, first one committed at university premises walking a path of 880 m in length. 

## 7. Conclusions

The results showed that the error distribution selected for foot-PDR placed too much emphasis on the rare but extreme errors and therefore the performance of the fusion was poor. When the fusion was conducted using the correct visual error distributions, but replacing the foot-PDR distributions with a Gaussian distribution, the resulting solution was suitable for infrastructure-free indoor localization. Because the methods are aimed to be used in tactical operations, a proof-of-concept experiment was conducted in a more realistic environment by soldiers experiencing motions typical for the application area. The results showed the feasibility of the developed method for infrastructure-free tactical localization. Our future research includes modelling of barometer and sonar measurement errors and their incorporation into the particle filtering fusion for three-dimensional localization solution. 

## Figures and Tables

**Table 1:** Most suitable measurement error distributions based on Chi-Square Goodness-of-Fit testing.

Error	Distribution	Parameters	Test Statistic	*p*-Value	Degrees-Of-Freedom
Visual heading	Logistic	0.004, 1.77	1.68	0.19	1
Visual speed	Extreme value	0.045, 0.39	7.63	0.27	6
Foot-PDR heading	t location-scale	−0.112, 3.25, 1.12	4.44	0.73	7
Foot-PDR speed	t location-scale	−0.04, 0.15, 1.41	2.63	0.45	3

**Table 2:** Errors in horizontal position solution.

Error Distribution	RMSE	CEP	90 Percentile
Modelled	20.9 m	28.9 m	56.3 m
Visual modelled	8.8 m	7.9 m	22.3 m
Gaussian, zero mean	16.3 m	25.8 m	44.7 m
Gaussian, computed means	8.8 m	9.7 m	23.1 m
